# Clinical application of red cell distribution width, mean platelet volume, and cancer antigen 125 detection in endometrial cancer

**DOI:** 10.1002/jcla.23309

**Published:** 2020-03-20

**Authors:** Hongyu Zhang, Ka Liang, Liuhua Ke, Shifu Tang

**Affiliations:** ^1^ Department of Clinical Laboratory The Third Affiliated Hospital of Guangxi University of Chinese Medicine Liuzhou Traditional Chinese Medical Hospital The Third Clinical Faculty of Guangxi University of Chinese Medicine Liuzhou China

**Keywords:** cancer antigen 125, endometrial cancer, endometrial hyperplasia, mean platelet volume, red cell distribution width

## Abstract

**Background:**

Red cell distribution width (RDW) and mean platelet volume (MPV) are considered to be associated with tumors. We investigated the diagnostic value of RDW, MPV, and cancer antigen 125 (CA125), alone or in combination, in the diagnosis of endometrial cancer and endometrial hyperplasia.

**Methods:**

This study included 144 patients with endometrial cancer (stage I: 32; II: 42; III: 48; and IV: 22), 104 patients with endometrial hyperplasia, and 80 healthy control subjects. The whole blood cell parameters were analyzed by a Mindray Blood Cell Analyzer (CAL8000), whereas CA125 was analyzed using an Architect i2000 Analyzer (Abbott).

**Results:**

Significant differences in RDW, MPV, and CA125 level were observed in the endometrial cancer, endometrial hyperplasia, and control groups (*P* < .05). Red cell distribution width was positively correlated (*r* = .735) whereas MPV was negatively correlated with (*r* = −.736) endometrial cancer staging. The area under the receiver operating characteristic curve of the combined diagnosis of endometrial cancer based on RDW, MPV, and CA125 was 0.924 (95% CI: 0.881‐0.955). The sensitivity and specificity of the combined diagnosis were larger than those of the independent detections involving RDW, MPV, and CA125.

**Conclusions:**

The combination of RDW, MPV, and CA125 can improve the differential diagnosis of endometrial cancer and endometrial hyperplasia.

## 1 INTRODUCTION

Endometrial cancer (EC) is the most common gynecological malignancy. It is the fourth most common cancer among American women after breast, lung, and colorectal cancers,^1^ and its incidence is expected to increase in the next 10 years. Early‐stage endometrial cancer (stage I) is usually cured by surgery; however, patients with advanced endometrial cancer (stage III or IV) have poor prognosis, and their 5‐year overall survival rates range from 47% to 69% (stage III) and from 15% to 17% (stage 4).^2^ Obesity is a risk factor for endometrial cancer; in fact, several bioactive molecules produced by adipose tissue, such as insulin‐like growth factors, insulin, sex steroids, and their activation signals, promote the progression of endometrial cancer.^3^ Most endometrial cancer cases are believed to be caused by excessive estrogen exposure due to the absence of the balancing effect of progesterone, inducing endometrial proliferation and subsequently endometrial hyperplasia and cancer.^4^

Endometrial hyperplasia (EH) is a common endocrine disease in women, and it is mainly characterized by irregular vaginal bleeding, infertility, and even malignant transformation. Endometrial atypical hyperplasia, which has a certain tendency to develop into cancer, is recognized as a precancerous lesion of endometrial cancer. In fact, 29% of untreated complex atypical hyperplasia develops into cancer, and 46% of preoperative patients have adenocarcinoma in their hysterectomy specimens.^5^ The development of inflammation is an important factor in the progression and promotion of the pathology of endometrial hyperplasia; it is also a risk factor for the progression of endometrial hyperplasia into malignant tumors.^6^

Hematological parameters in routine blood tests are considered inflammatory markers. Red blood cell distribution width (RDW) is an important indicator of consistency in size of red blood cells, and mean platelet volume (MPV) is the main parameter used to assess platelet activation. Red cell distribution width and MPV play an important role in cancer progression, and they are associated with tumor stage and metastasis; for instance, they are associated with poor tumor prognosis in esophageal squamous cell carcinoma^7^ and breast cancer.^8^ However, the role of these parameters in endometrial cancer and endometrial hyperplasia has not yet been fully understood; therefore, this study aimed to investigate the role of RDW, MPV, and serum cancer antigen (CA) 125 (alone or in combination) in the diagnosis of endometrial cancer and endometrial hyperplasia.

## 2 METHODS

### 2.1 Patients

We performed a retrospective study involving patients with endometrial cancer diagnosed at the Liuzhou Traditional Chinese Medical Hospital, China, from December 2017 to August 2019. The patients included in this study were pathologically diagnosed with endometrial cancer, and they did not receive any treatment before diagnosis. Blood samples were taken from the patients with the complaint of abnormal uterine bleeding, and all samples were taken before the endometrial biopsy performed.^9^ The following patients were excluded: those with blood disease, diabetes mellitus, kidney disease, acute inflammation, anemia, and cardiovascular disease; those who have recently undergone iron therapy and blood transfusion (within the last 3 months); and those with venous thrombosis for >6 months. A total of 144 patients with endometrial cancer were included (stage I: 32; II: 42; III: 48; and IV: 22). These patients were classified into groups in accordance with the standards established by the International Federation of Gynecology and Obstetrics in 2014. The endometrial hyperplasia group consisted of 104 patients diagnosed with endometrial hyperplasia in our hospital during the same time period. Endometrial hyperplasia was diagnosed through histological analysis. The control group consisted of 80 healthy people with no abnormal physical examination findings. This study was approved by the ethics committee of the Liuzhou Traditional Chinese Medical Hospital, China.

### 2.2 Method

Venous blood samples (2 mL) were obtained from all subjects in the morning and placed in EDTA‐K2 anticoagulation tubes and drying tubes. Whole blood cell parameters were determined using a CAL8000 Automated Hematology Analyzer (Mindray). White blood cell count, absolute neutrophil count, absolute lymphocyte count, hemoglobin concentration, blood platelet count, platelet distribution width (PDW), and red blood cell distribution width (RDW) were obtained directly by the hematology analyzer. In our hospital, the RDW ranged from 11.0% to 14.0%. CA125 concentrations were measured using an ARCHITECT analyzer and its commercial kit (Abbott Diagnostics). The CA125 cutoff value was 35 U/mL according to the manufacturer.

### 2.3 Statistical analysis

Data were analyzed using SPSS version 23.0 (IBM, Armonk, NY). Continuous variables are presented as mean ± standard deviation, non‐normally distributed data are expressed as median and quartile, and categorical variables are expressed as whole numbers and percentage. One‐way ANOVA was used to evaluate the differences in baseline data of the three groups. Tukey's test was performed to compare the indicator‐related differences between two groups. Correlations of cancer stage with RDW and MPV in endometrial cancer patients were analyzed by Spearman's correlation. The areas under curve (AUCs) were measured using the MedCalc Statistical Software (MedCalc Software bvba, Ostend, Belgium), which can reveal the sensitivity and specificity of a single diagnosis or a combined diagnosis. ROC curves were compared to test the statistical significance of the difference between areas. In all statistical tests, *P*‐values of <.05 (two‐tailed) indicated statistical significance.

## 3 RESULTS

A total of 144 patients with endometrial cancer (age range: 24‐77 years) were included in this study. According to the classification system of the International Federation of Gynecology and Obstetrics, 32 (22.2%), 42 (29.2%), 48 (33.3%), and 22 (15.3%) patients had stage I‐IV endometrial cancer, respectively. A total of 104 patients with endometrial hyperplasia (age range: 28‐62 years) and 80 healthy control subjects (age range: 22‐62 years) were also included in this study. These three groups did not significantly differ in terms of white blood cell count, absolute neutrophil count, absolute lymphocyte count, and platelet count. In terms of hemoglobin levels, a significant difference was observed between the endometrial cancer group and the endometrial hyperplasia group (*P* < .01) but not between the endometrial hyperplasia group and the control group (*P* > .05). Compared with the PDW measurements in the control group, that in the endometrial cancer group and in the endometrial hyperplasia group significantly increased; however, no significant difference in PDW measurement was observed between the endometrial cancer group and the endometrial hyperplasia group (*P* > .05). Moreover, significant differences were observed among the three groups in terms of RDW, MPV, and CA125 level (Table 1).

**TABLE 1:** Clinical characteristics of the participant

Characteristics	Endometrial cancer	Endometrial hyperplasia group	Controls group	*P*
Number	144	103	80	
Age (y)	46.33 ± 12.97	45.02 ± 10.88	44.96 ± 7.53	.441
W, ×10^9^/L	6.80 ± 2.21	6.67 ± 2.08	6.72 ± 2.33	.257
N, ×10^9^/L	4.56 ± 1.82	4.46 ± 1.77	4.62 ± 1.97	.185
L, ×10^9^/L	1.83 ± 0.92	1.68 ± 0.83	1.75 ± 0.84	.113
Hb, g/L	102.31 ± 24.62^a^	127.28 ± 18.91	131.02 ± 16.22^c^	<.001
RDW, %	15.06 ± 2.30^a^	14.17 ± 2.27^b^	12.76 ± 1.07^c^	<.001
PLT, ×10^12^/L	232 ± 98.33	241 ± 102.36	224 ± 95.23	.174
MPV; (fl)	12.01 ± 2.68^a^	11.51 ± 2.22^b^	9.58 ± 1.84^c^	<.001
PDW, %	17.02 ± 3.21	16.88 ± 2.89^b^	15.89 ± 1.46^c^	<.001
CA125; U/mL	39.06 ± 33.94^a^	32.87 ± 10.77^b^	17.50 ± 8.35^c^	<.001

Note

*P‐*values were calculated by one‐way ANOVA tests.

Abbreviations: CA125, cancer antigen 125; Hb, hemoglobin; L, absolute lymphocyte count; MPV, mean platelet volume; N, absolute neutrophil count; PDW, platelet distribution width; PLT, blood platelet count; RDW, red cell distribution width; W, white blood cell count.

^a^ Shows a significant difference (*P* < .05) between endometrial cancer group and endometrial hyperplasia group (Tukey's test).

^b^ Shows a significant difference (*P* < .05) between endometrial hyperplasia group and controls group (Tukey's test).

^c^ Shows a significant difference (*P* < .05) between endometrial cancer group and controls group (Tukey's test).

**FIGURE 1:**
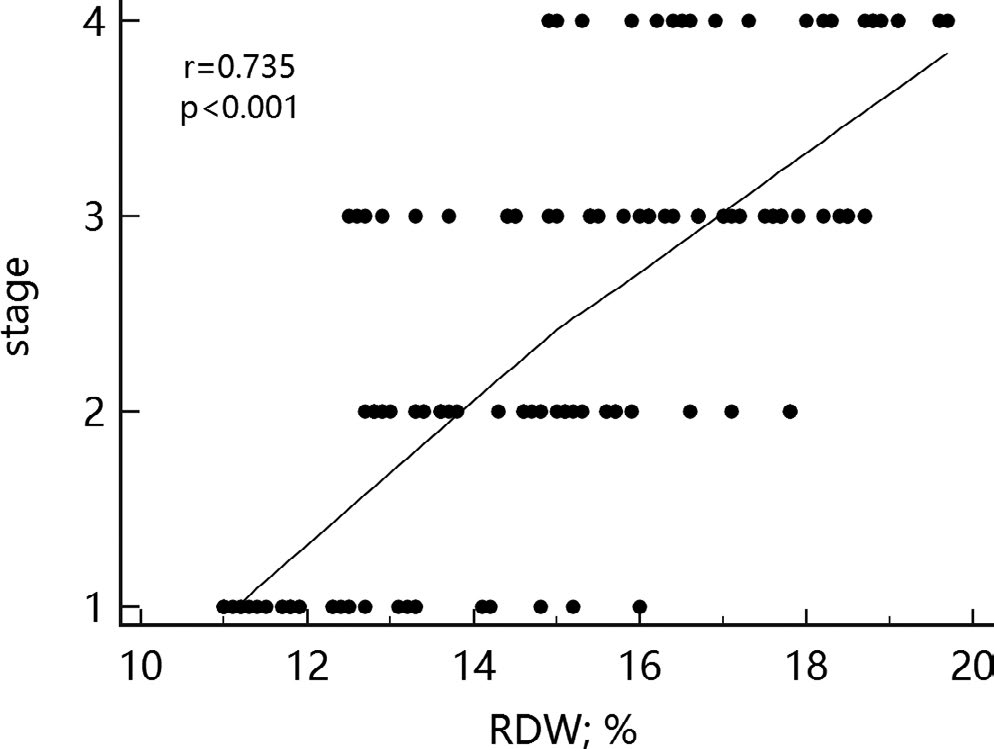
Correlation between red cell distribution width and cancer stage

**FIGURE 2:**
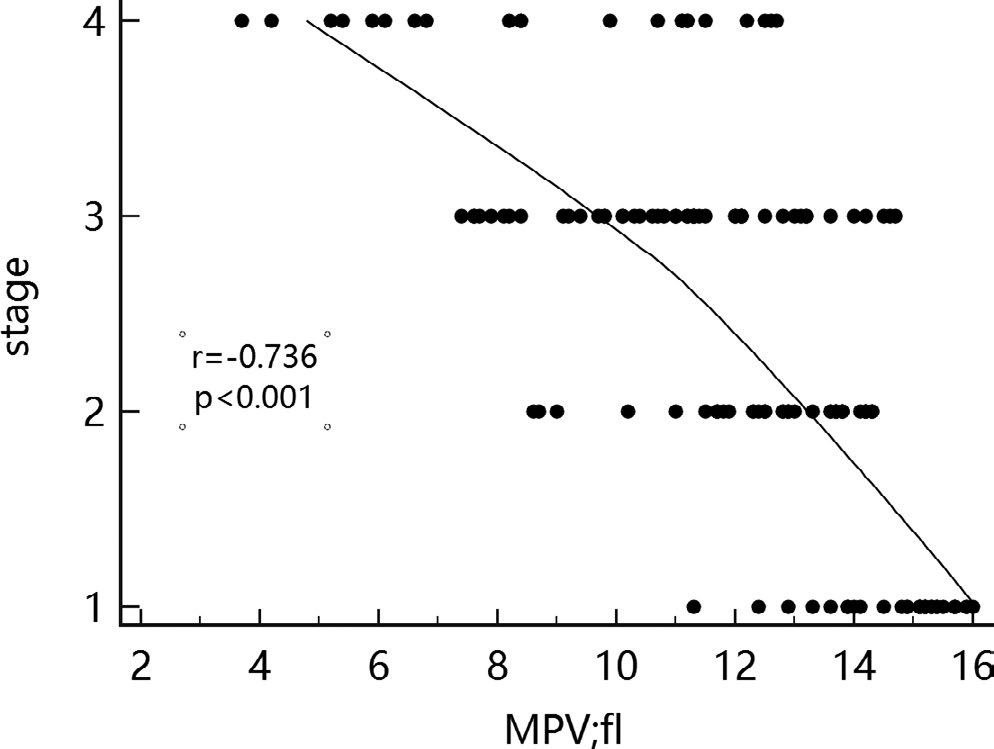
Correlation between mean platelet volume and cancer stage

Correlation analysis results showed that RDW in endometrial cancer patients was positively correlated with cancer stage, whereas MPV was negatively associated with cancer stage (Figures 1 and 2).

**FIGURE 3:**
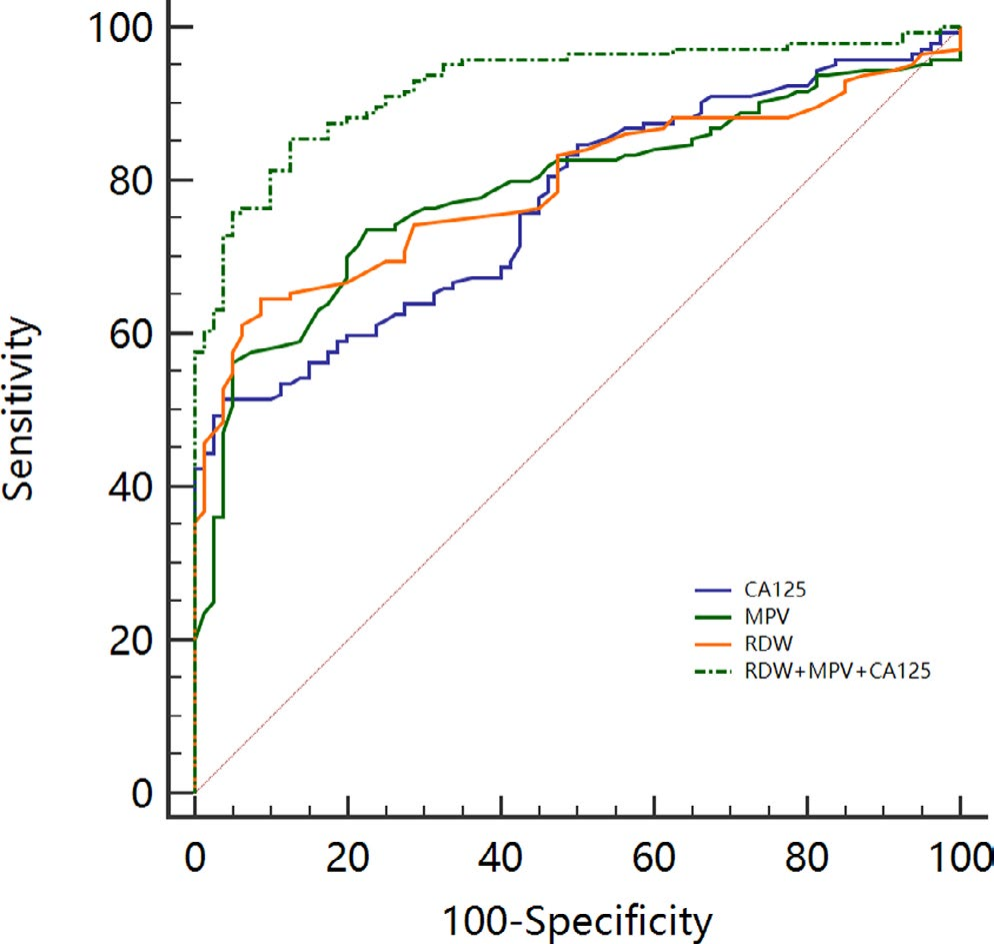
ROC curve plot for endometrial cancer

The ROC curve was used to analyze the diagnostic value of MPV, RDW, CA125, and their combination to diagnose endometrial cancer. The results are shown in Figure 3 and Table 2. The AUC values for endometrial cancer diagnosis based on RDW, MPV, and CA125 were 0.792 (0.733‐0.843), 0.787 (0.727‐0.838), and 0.768 (0.708‐0.822), respectively. Among the three parameters, RDW had higher diagnostic sensitivity than MPV and CA125 (64.6% vs 56.3% and 51.4%), whereas CA125 had higher diagnostic specificity than RDW and CA125 (81.3% vs 78.8% and 76.3%). The combined detection involving these three parameters showed the largest AUC for endometrial cancer diagnosis (0.924, 0.881‐0.955). In addition, compared with the sensitivity and specificity of the three indicators for the independent diagnosis of endometrial cancer, the sensitivity and specificity of the combined diagnosis are higher (86.8% and 88.8%, respectively). Pairwise comparison of the ROC curves indicated that the AUC of the combined diagnosis was significantly larger than that of the three indicators (Table 3).

**TABLE 2:** Predictive efficiency of each marker for diagnosis of endometrial cancer

Markers	AUC (95% CI)	Sensitivity (%)	Specificity (%)	+PV	−PV
RDW	0.792 (0.733‐0.843)	64.6	78.8	84.5	55.3
MPV	0.787 (0.727‐0.838)	56.3	76.3	81.0	49.2
CA125	0.768 (0.708‐0.822)	51.4	81.3	83.1	48.1
RDW + MPV + CA125	0.924 (0.881‐0.955)	86.8	88.8	93.3	78.9

Abbreviations: +PV, positive predictive value; AUC, area under curve; CI, confidence interval;−PV, negative predictive value.

**TABLE 3:** Pairwise comparison of ROC curves

	CA125 vs MPV	CA125 vs RDW	CA125 vs combine	MPV vs RDW	MPV vs combine	RDW vs combine
Difference between areas	0.018	0.024	0.156	0.006	0.138	0.132
Standard error	0.045	0.043	0.030	0.042	0.026	0.027
95% confidence interval	(−0.070 to 0.11)	(−0.060 to 0.107)	(0.098 to 0.213)	(−0.076 to 0.087)	(0.086 to 0.189)	(0.079 to 0.185)
*Z* statistic	0.401	0.550	5.279	0.134	5.240	4.917
*P*	.688	.582	<.001	.894	<.001	<.001

Note

Combine: RDW + MPV + CA125.

## 4 DISCUSSION

Many studies have already reported on the identification of endometrial cancer and endometrial hyperplasia and on the diagnosis of endometrial cancer. The use of many new markers or some scoring systems has been instrumental in the identification and diagnosis of endometrial cancer and endometrial hyperplasia.^10^, ^11^ In this study, blood parameters of the endometrial cancer group and of the endometrial hyperplasia group were investigated. Significant differences in RDW, MPV, and CA125 level were observed in the endometrial cancer, endometrial hyperplasia, and control groups (*P* < .05). Moreover, RDW was positively correlated (*r* = .735) whereas MPV was negatively correlated (*r* = −.736) with endometrial cancer staging. ROC curve analysis demonstrated the efficiency of the combined detection of endometrial cancer based on RDW, MPV, and CA125. No similar finding has been reported in the literature.

The mechanism of action of RDW on tumors is mainly manifested in inflammation, poor nutritional status, and oxidative stress. First, malignant tumors are usually accompanied by systemic inflammatory reactions, and RDW is considered to be a new marker of inflammation. Red cell distribution width is positively correlated with tumor necrosis factor alpha (TNF‐alpha) and interleukin (IL)‐6 in rheumatoid arthritis, suggesting that RDW is a potential adjunct marker that reflects an inflammatory process.^12^ In addition, it has been reported in the literature that RDW is a sensitive marker of inflammation and is an important and independent predictor of low survival rate in patients with esophageal squamous cell carcinoma after therapeutic esophagectomy.^13^ Therefore, the claim that RDW can reflect the state of inflammation in cancer is reasonable. Second, weight loss and malnutrition are other signs of cancer. Malnutrition is caused by a deficiency in vitamins and minerals (ie, folic acid, vitamins B6, B12, A, C, E, D, riboflavin, iron, and zinc)^14^; however, folic acid and vitamin B12 affect DNA synthesis, that is, they slow down erythropoiesis, causing the RDW in megaloblastic anemia to increase. Last, considerable changes in red blood cell volume are strongly associated with a decrease in red blood cell deformability, which in turn impairs blood flow through the microcirculation; as a result, the function of the microcirculation is impaired and the peripheral tissue oxygenation is reduced, promoting sustained inflammation and oxidative stress.^15^ In our study, RDW increased with endometrial cancer staging. Possibly, with the progression of endometrial cancer, the inflammation, malnutrition, and oxidative stress are becoming worse.

The MPV is a parameter calculated and provided by automatic hematology analyzer. Through a systematic review and meta‐analysis, Jung‐Soo et al^16^ found that MPV is significantly higher in individuals with various malignancies than in healthy individuals. Moreover, Stojkovic et al^17^ found that MPV did not significantly differ between early (I and II) and advanced (III and IV) disease stages, but a decreasing trend was observed in TNM stages I‐IV (*P* = .662). These conclusions are basically consistent with our findings. The increased MPV in tumors may be related to inflammation. Dossus et al^18^ conducted a nested case‐control study of patients with endometrial cancer to determine the risk of endometrial cancer, and they found that the inflammation factor cytokines (especially IL‐6, TNF receptor, and C peptide) had an odds ratio of 1.62 for developing endometrial cancer. Elevated IL‐6 levels are observed in almost all types of tumors, and they promote tumorigenesis by regulating apoptosis, survival, proliferation, angiogenesis, metastasis, and metabolism.^19^ In turn, tumors promote platelet production and activation via the IL‐6 pathway. Megakaryocytic maturation, platelet production, and platelet size are regulated by cytokines, such as macrophage colony‐stimulating factor, granulocyte colony‐stimulating factor, and IL‐6.^20^ Therefore, the increase in MPV may be related to cancer occurrence and progression.

CA125 is a relatively classic tumor marker and is often used in combination with epididymal protein 4 to improve the effectiveness of ovarian cancer diagnosis. CA125 plays an important role in the differential diagnosis of abnormal uterine bleeding and endometrial cancer.^21^ Wang et al^22^ combined the use of the serum tumor markers carcinoembryonic antigen, CA15‐3, CA125, CA19‐9, and tissue polypeptide‐specific antigen to improve the efficiency of metastatic breast cancer diagnosis. Our study shows that CA125 combined with RDW and MPV increases the AUC and improves the diagnostic efficiency.

There are some limitations in our research. This retrospective study on endometrial cancer and endometrial hyperplasia involved a relatively small sample size. Therefore, a large‐scale, multi‐center, forward‐looking study is needed to validate our conclusions. In addition, this study included Chinese participants only, so our conclusions are not applicable to other populations. Nonetheless, this study was the first to determine the clinical value of RDW, MPV, and CA125 in endometrial cancer and endometrial hyperplasia and in their combined use for improved efficiency of endometrial cancer diagnosis.

## CONFLICT OF INTEREST

All authors declare that they have no conflict of interest.

## Data Availability

Some or all data used during the study are available.
